# Colonoscopy is the first choice for early postoperative rectal anastomotic bleeding

**DOI:** 10.1186/1477-7819-12-376

**Published:** 2014-12-06

**Authors:** Zheng Lou, Wei Zhang, Enda Yu, Ronggui Meng, Chuangang Fu

**Affiliations:** Department of Colorectal Surgery, Changhai Hospital, Changhai Road 168#, 200433 Shanghai, China

**Keywords:** Anastomotic bleeding, Rectal cancer, Colonoscopy, Hemostasis

## Abstract

**Background:**

Anastomotic bleeding is rare but is one of the dangerous complications, with associated morbidity and mortality, at the early stage of rectal cancer surgery. The aim of this study was to report our experiences in the treatment of this emergency condition.

**Methods:**

We retrospectively analyzed the general characteristics, treatment and outcome of patients with severe anastomotic bleeding after undergoing rectal cancer resection with stapled anastomosis at the Department of Colorectal Surgery of Changhai Hospital (China) between January 2011 and December 2013.

**Results:**

Anastomotic bleeding occurred in six out of 2,181 patients (0.3%) who underwent anterior resection with stapled anastomosis due to rectal cancer. All patients’ bleeding was stopped with colonoscopic techniques. There were no anastomotic leakages or strictures in these six patients.

**Conclusions:**

Anastomotic bleeding was a very rare complication after rectal cancer resection with stapled anastomosis. Colonoscopic treatment, including electrocoagulation and clipping, were both safely and effectively used in the early postoperative period to cease persistent anastomotic bleeding.

## Background

Anastomotic bleeding is one of the dangerous complications, with associated morbidity and mortality, at the early postoperative stage of rectal cancer surgery. Although uncommon, significant anastomotic bleeding after rectal resection can be severe enough to require re-operation. This emergency has been reported in recent years [1–3]. However, there are only a few clinical reports focused on the treatments of anastomotic bleeding because of the limited number of cases. The aim of the present study was to report our experiences of colonoscopic hemostasis of anastomotic bleeding at the early postoperative stage after anterior resection for rectal cancer.

## Methods

The ethical committee of Changhai hospital provided ethical approval for this study. Changhai Hospital (Shanghai, China) is a large tertiary hospital that includes medical practice, teaching and scientific research. About 1,500 colorectal cancer patients receive surgical treatment per year. In the present study we included patients from our department between January 2011 and December 2013 who had persistent bleeding of the rectum after rectal anastomosis within the first four postoperative weeks, and one or more of the following criteria as previous report [4]: a significant fall in hemoglobin, need for blood transfusion, hemodynamic instability or shock and, finally, the need for any emergency intervention such as colonoscopy or surgery. All patients received a standardized operation regimen and perioperative medicine according to the hospital protocol. Clinical data concerning demographics, risk factors of hemorrhage, location of the tumor (distance to the anal verge), type of procedure, protective ileostomy, time to postoperative bleeding, colonoscopic hemostatic techniques, complications and postoperative stay were collected retrospectively. A colonoscope (CV-260sl, Olympus, Tokyo, Japan) was used to check for anastomotic bleeding. We used clips (HX-610-135 l, Olympus, Tokyo, Japan) and a high frequency electric surgical operation system (VIO 200 S, ERBE Elektromedizin GmbH, Tubingen, Germany) for hemostasis.

### Strategies of colonoscopic hemostasis

Bowel preparations were not required before the colonoscopic hemostasis. Blood and clots often masked the bleeding points, therefore, the first step is to fill the intestinal lumen with cold water to make it possible to identify the bleeding points. There were two different hemostatic strategies for different anastomotic bleeding types. If a colonoscopy revealed an active and continuous bleeding point from the artery, clipping would be adopted. If bleeding was caused by mucosal staxis, electrocoagulation would be adopted.

## Results and discussion

Between January 2011 and December 2013, 2,181 patients underwent anterior resection with stapled anastomosis due to rectal cancer. In six out of 2,181 (0.3%) patients, anastomotic bleeding that started within seven days of surgery required further intervention than pharmaceutical treatment alone. All of these six patients underwent a low anterior resection. A colonoscopy was undertaken and bleeding was controlled by electrocoagulation or clipping. One out of the six patients experienced a reoccurrence of anastomotic bleeding after the third day of the first postoperative colonoscopy. The patient received colonoscopic electrocoagulation again and the anastomotic bleeding stopped. The characteristics of these six patients and their treatment are shown in Table 1. No patient suffered an immediate or delayed complications related to their anastomosis following colonoscopic treatment, with a minimum of a 10-month follow-up period.

**Table 1:** **Characteristics, treatments and results of seven patients**

Number	Age	Sex	Comorbidity	DAV (cm)	Type of procedure	Protective ileostomy	Time to postoperative bleeding	Hemoglobin drop (g/dl)	Blood pressure (mmHg)	Blood transfusion (units)	Colonoscopic hemostatic techniques	Complication	Postoperative stay (days)
1	64	Male	None	3	Open	Yes	4th day	3.1	150/90	None	Electrocoagulation	No	8
2	46	Female	None	6	Laparoscopy	Yes	2 hours	3.6	118/86	4 RBCs	Clipping	No	12
										2 Plasma			
3	64	Male	Hypertension	5	Laparoscopy	Yes	6.5 hours	6.6	90/50	9 RBCs	Clipping	No	7
										6 Plasma			
4	72	Female	Hypertension	6	Open	Yes	7th day	3.7	110/68	None	Electrocoagulation	No	10
5	50	Female	Hypertension	4	Open	Yes	24 hours (First time)	2.5	118/72	2 RBCs	Electrocoagulation	Bleeding again	20
							4th day (Second time)			1 Plasma	Electrocoagulation	No	
6	61	Male	None	8	Open	Yes	11 hours	4.1	140/81	1 RBCs	Clipping	No	16
										2 Plasma			

DAV = distance to the anal verge.

Continuous rectal anastomotic bleeding is rare. However when it occurs, it may need further intervention. In this study, we reported our experiences of successful colonoscopic hemostasis for rectal anastomotic bleeding at the early postoperative stage. All patients avoided more invasive operations for hemostasis and no colonoscopic complications occurred.

The incidence of postoperative colorectal anastomotic bleeding varies from 0.4 to 4% [5, 6]. In our study, the incidence of stapled anastomotic bleeding was 0.3%, lower than that reported in previous reports. Staple line reinforcement has been postulated to reduce associated bleeding risk [7]. Our lower incidence may be attributed to the technique of whole layer interrupted reinforcement sutures after stapled anastomosis.

There are some special characteristics in anastomotic bleeding after stapled rectal anastomosis, such as repeated defecation with blood or blood clots and rectal irritation symptoms. Anastomotic bleeding is usually treated successfully by drugs alone. However, more invasive methods such as colonoscopy or even reoperation are occasionally necessary as anastomotic bleeding can be severe enough to cause hemorrhagic shock. This kind of emergency commonly occurs in the early postoperative period. The time interval to postoperative hemorrhage from a stapled colorectal anastomosis is reported to range from four hours to nine days [8]. In the present study, anastomotic bleeding occurred between two hours and seven days. It is very important to carry out close postoperative monitoring in order to detect rectal anastomotic bleeding as early as possible.

Colonoscopy allows for a direct inspection of the anastomosis with subsequent application of various means of hemostasis. Furthermore, this method has several advantages. Firstly, colonoscopy exerts less physiological stress on patients compared with reoperation. Secondly, no anesthesia is necessary, although some patients may need to have a mild sedative. Thirdly, reoperation and associated complications are thereby avoided. Finally, it is cost-saving in both operative costs and overall length of hospital stay.

However, there are two limitations of this intervention. Firstly, colonoscopy may seem aggressive and possibly even dangerous to perform in the early postoperative period because of air insufflation, local colonoscope trauma and torque [9]. Thus colonoscopic hemostasis needs to be performed by skilled and experienced doctors. Another limitation is the possibility of anastomotic disruption and subsequent leakage due to colonoscopic electrocoagulation at the early postoperative stage. Cirocco and Golub reported the successful use of electrocoagulation, although they noted that an anastomotic fistula that developed in one of their six cases might have been related to this technique [10]. However, due to the small number of cases reported there is not sufficient statistical evidence to demonstrate an increased risk of anastomotic fistula with colonoscopic electrocoagulation. In the present study, we adopted colonoscopic electrocoagulation or clipping of the anastomotic bleeding, and none of our patients developed anastomotic fistula. It remains difficult to know if the beginning of this troublesome complication is the bleeding itself, its hemodynamic consequences or the treatment of the anastomosis [2].

Anastomotic site, surgical methods and protective colostomy have been reported to be associated with anastomotic bleeding [11]. However, the role of many factors remains controversial. So far, the risk factors for anastomotic bleeding have not been fully elucidated, and anastomotic bleeding cannot be accurately predicted and prevented with precise and effective methods. Our results suggest that anastomotic bleeding appears more likely after anus-preserving operations for low rectal cancer. Some authors suggest adopting an intraoperative colonoscopy in order to routinely secure stapling colorectal anastomosis [2]. However, it is usually impossible to adopt a routine colonoscopy procedure in this manner to check anastomosis perfection in many hospitals. We propose that intraoperative colonoscopy should be adopted for anastomotic bleeding only in those in whom bleeding is suspected during surgery.

## Conclusions

Rectal anastomotic bleeding is a rare complication in the early postoperative stage. When it occurs, colonoscopy may be the first choice of hemostasis. Colonoscopic techniques, including electrocoagulation and clipping, are both a safe and effective way to control anastomotic bleeding.
